# Genomics for greater efficiency in pigeonpea hybrid breeding

**DOI:** 10.3389/fpls.2015.00793

**Published:** 2015-10-01

**Authors:** Rachit K. Saxena, K. B. Saxena, Lekha T. Pazhamala, Kishan Patel, Swathi Parupalli, C. V. Sameerkumar, Rajeev K. Varshney

**Affiliations:** International Crops Research Institute for the Semi-Arid Tropics, Patancheru, India

**Keywords:** pigeonpea, hybrids, markers, purity assessment, fertility restorers

## Abstract

Cytoplasmic genic male sterility (CGMS) based hybrid technology has demonstrated its immense potential in increasing the productivity of various crops, including pigeonpea. This technology has shown promise for breaking the long-standing yield stagnation in pigeonpea. There are difficulties in commercial hybrid seed production due to non-availability of field-oriented technologies such as time-bound assessment of genetic purity of hybrid seeds. Besides this, there are other routine breeding activities which are labor oriented and need more resources. These include breeding and maintenance of new fertility restorers and maintainer lines, diversification of cytoplasm, and incorporation of biotic and abiotic stress resistances. The recent progress in genomics research could accelerate the existing traditional efforts to strengthen the hybrid breeding technology. Marker based seed purity assessment, identification of heterotic groups; selection of new fertility restorers are few areas which have already been initiated. In this paper efforts have been made to identify critical areas and opportunities where genomics can play a leading role and assist breeders in accelerating various activities related to breeding and commercialization of pigeonpea hybrids.

## Introduction

**FIGURE 1:**
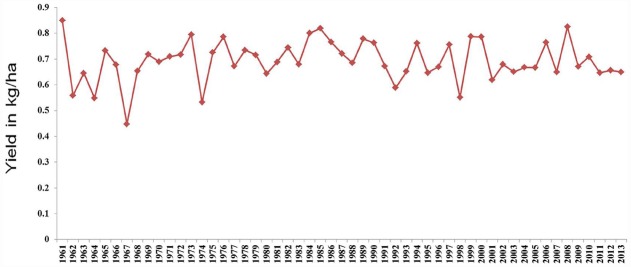
**Trends in pigeonpea yield from year 1961 to year 2013**.

**FIGURE 2:**
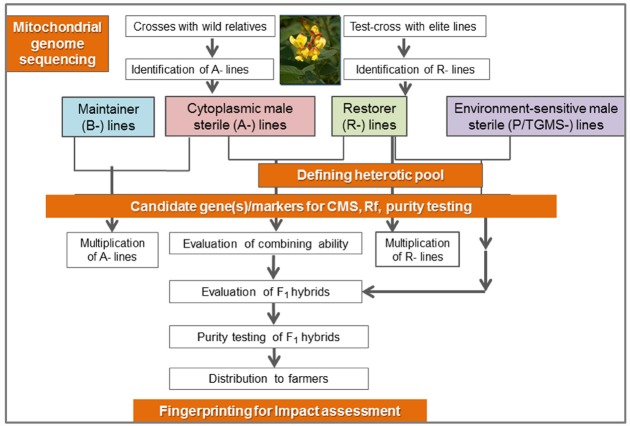
**Schematic representation of genomics initiatives for enriching hybrid breeding in pigeonpea**.

Pigeonpea [*Cajanus cajan* (L.) Millspaugh] is an important pulse crop of rainfed and semi-arid regions of Asia, Africa and the Caribbean islands. India accounts for over 85% of the global area of 4.6 million hectares (Table 1). Globally, it is grown under low input environments, primarily as an intercrop with early maturing cereals. Pigeonpea is a major protein supplement for small holding farming families. Pigeonpea is also known for improving soil nutrition by fixing atmospheric nitrogen, releasing soil-bound phosphorus and recycling micro-nutrients. Its extensive root mass and leaf fall are known for improving soil structure and water infiltration in the soil (Saxena, 2008). Pigeonpea varietal improvement program started in India in 1931 with selection from landraces for traits such as seed size, fusarium wilt, plant type, and yield (Ramanujam and Singh, 1981). During this period over 100 pigeonpea cultivars have been released in India (http://www.iipr.res.in/aicrp.html); but the crop productivity remained stagnant (Figure 1). This is a matter of concern in view of increasing population and reducing per capita availability of protein that led to malnutrition among growing children and women, in particular. Considering the above mentioned constraints, new scientific approaches and tools are needed to raise the productivity of this important pulse crop. In this context, cytoplasmic male sterility (CMS)-based hybrid technology was developed and the world’s first pigeonpea commercial hybrid namely; ICPH 2671 was released, with 46% yield advantage in farmers’ field (Saxena et al., 2013). This is considered a milestone in the history of pulse breeding so far. In order to popularize hybrids, it is necessary that new high yielding hybrids are bred for different climatic conditions. Besides this, the technology be made grower-friendly. In this regard the new developments in genomics science can be of great help. Beyond doubt, integration of genomics with breeding can enhance the pace of breeding new widely adopted hybrids. The genomics science can be effectively used in the selection of heterotic hybrid male and female parents, incorporation of resistances and stability in the performance, assessment of purity of hybrids and their parents. In this paper, an effort has been made to highlight the potential role that genomics can play in accelerating the pace of hybrid breeding in pigeonpea (Figure 2).

**TABLE 1:** **Pigeonpea cultivation area and production in different countries.**.

**Country**	**Area harvested (Ha)**	**Production (tons)**
Bahamas	135	180
Bangladesh	500	423
Burundi	4,786	7,386
Myanmar	650,000	800,000
Comoros	500	430
Dominican Republic	24,103.21	26,855.12
Grenada	550	800
Haiti	108,633.63	86,906.91
India	4,650,000	3,022,700
Jamaica	832	957
Kenya	144,218	73,183
Malawi	217,068	287,983
Nepal	17,459	16,459
Panama	3,800	1,970
Philippines	514	858
Puerto Rico	360	320
Saint Vincent and the Grenadines	20	210
United Republic of Tanzania	287,182	247,387
Trinidad and Tobago	915	770
Uganda	105,000	93,930
Venezuela	4,286.35	3,227.63
Democratic Republic of the Congo	11,000	7,000

## Overview of Hybrid Breeding in Pigeonpea

Pigeonpea is unique among the pulses as its floral morphology allows partial cross-pollinations (Saxena et al., 1990). International Crops Research Institute for the Semi-Arid Tropics (ICRISAT) in 1974 started breeding hybrids using the natural out-crossing. As a first step a program was launched for breeding a male sterility system that could be used in breeding hybrids; and a genetic male sterility (GMS) system, controlled by a single recessive gene (*ms*_1_*ms*_1_), was identified (Reddy et al., 1978). This GMS was used to develop hybrid technology to assess the extent of hybrid vigor and ability of out-crossing in seed production on the male sterile plants. The first ever pigeonpea hybrid ICPH 8 performed very well in the multi-location trials, coordinated trials, and in the farmers’ fields with mean standard heterosis of 25–35%, was released in 1991 for cultivation (Saxena et al., 1992). This was followed by the release of five other GMS based hybrids bred at different centers of Indian Council of Agricultural Research (ICAR; Saxena et al., 2006). Despite the yield advantages of 25–40%, these hybrids could not be commercialized due to seed production difficulties (Saxena et al., 2006). This valuable experience indicated that in pigeonpea sufficient heterosis is available and seed production issues can be tackled economically if the GMS system could be replaced with cytoplasmic genic male sterility (CGMS) system. Any hybrid technology that is based on CGMS system, works on three different plant genetic systems and therefore it is popularly known as a “three line hybrid system.” This essentially includes male-sterile (A- line); its maintainer (B- line), and restorer (R- line). The early efforts to breed a good CGMS did not succeed (Reddy and Faris, 1981; Ariyanayagam et al., 1995; Tikka et al., 1997). The real breakthrough came when Saxena et al. (2005) developed a CGMS system by crossing a wild species of pigeonpea (*Cajanus cajanifolius*) as a female parent with a cultivar as male parent. This male sterility system was found ideal for hybrid breeding and was designated as A_4_ CMS. The genetics of fertility restoration of A_4_ cytoplasm was studied and two dominant genes were found controlling the fertility of the hybrids (Dalvi et al., 2008; Kyu et al., 2011; Sawargaonkar et al., 2012).

### Commercial Pigeonpea Hybrids

The first commercial pigeonpea hybrid ICPH 2671, produced by crossing ICPA 2043 with ICPR 2671, was released in 2010 (Saxena et al., 2013). In 1,829 on-farm trials (Table 2) conducted in states of Maharashtra (782 trials), Andhra Pradesh (399 trials), Madhya Pradesh (360 trials), and Jharkhand (288 trials), ICPH 2671 recorded 35–69% superiority over the best local cultivars. Overall, in all the four states, ICPH 2671 was 51% better than the control in its productivity. After the success of hybrid ICPH 2671 in Madhya Pradesh, two more medium duration hybrids with high yield potential were released in India. In 2012, ICPH 2740 was released for cultivation in Andhra Pradesh (Saxena and Tikle, 2015); while the third hybrid ICPH 3762 was released in Odisha in 2014 (Saxena et al., 2014a). Like ICPH 2671, the hybrids ICPH 2740 and ICPH 3762 also out-yielded the control by a big (40–50%) margin. The performance data of the hybrid have shown that in pigeonpea significantly high productivity levels can be achieved by farmers and the persistent yield plateau can be cracked.

**TABLE 2:** **Performance of the commercial hybrid ICPH 2671 in on-farm trials**.

**State of India**	**No of farmers**	**Hybrid yield (kg/ha)**	**Control yield (kg/ha)**	**Standard heterosis (%)**
Maharashtra	782	969	717	35
Andhra Pradesh	399	1,411	907	55
Jharkhand	288	1,460	864	69
Madhya Pradesh	360	1,940	1,326	46
Total/mean	1,829	1,445	954	51

Source: Saxena et al. (2013).

### Constraints in Breeding New Hybrids

The major constraints in pigeonpea hybrid breeding as recognized now are (i) long generation turnover time that slow down the breeding and selection speed, (ii) determination of genetic diversity is another factor that limits selection of heterotic hybrid parents, and (iii) the on-farm seed production exercise. This also showed that it is possible to produce the required amount of seed with ease but its genetic purity may be compromised by some growers, hence a solution for this is a must in the near future to keep the ball rolling in the right direction. To deliver the benefits of hybrid technology to farmers, it is imperative that the process of breeding new hybrids be enhanced and seed technology is simplified. It is envisaged that the new developments in genomics science can help in solving these issues.

## Genomics for Accelerated Hybrid Breeding Program

During recent years, various genomic resources have been developed including a draft of the nuclear and the complete mitochondrial genome sequence in pigeonpea (Varshney et al., 2012; Tuteja et al., 2013), in addition to the large repertoire of molecular markers (Saxena et al., 2014b), high throughput genotyping platforms (Varshney et al., 2010), transcriptome assembly (Kudapa et al., 2014) and genetic maps (Saxena et al., 2010b; Bohra et al., 2012). As a result of the recent advances in the next generation sequencing (NGS) based approaches, large numbers of molecular markers including SSRs (>54,000; simple sequence repeat), ISRs (>29,000; intron spanning region), and SNPs (>12,000; single nucleotide polymorphism) in addition to the 25,577 ESTs have become available in the public domain (Pazhamala et al., 2015). Using genotyping by sequencing (GBS) and whole genome re-sequencing (WGRS) approaches, more and more variations such as SNPs, INDELs, CNVs, and PAVs are being identified which will be available and utilized in the near future for pigeonpea improvement programs. Apart from these resources, many inter- and intra-specific genetic maps were developed, some of which utilized mapping populations with CMS lines segregating for fertility restoration (Saxena et al., 2011). All these genomic resources along with the recent approaches will be utilized for consolidation and strengthening of the pigeonpea hybrid breeding technology in the near future. Furthermore, a number of efforts have been initiated toward rapid solutions to hybrid production systems and unlocking the mystery of heterosis using various genomics approach. Additionally, efforts have also been directed toward diversification of CMS sources in the pigeonpea genepool and answering the biological questions such as molecular basis of cytoplasmic male sterility. Also, various sets of molecular markers have been identified for fertility restoration and purity assessment. In the following sections we have provided details on accomplished, ongoing and future genomics efforts in pigeonpea hybrid breeding program (Figure 2).

### Molecular Basis of CMS

For sustainable pigeonpea hybrid production, diversification of CMS sources is highly required. At present there are eight cytoplasmic sources available (Ariyanayagam et al., 1995; Saxena and Kumar, 2003; Wanjari et al., 1999; Mallikarjuna and Saxena, 2005; Saxena et al., 2005; Mallikarjuna et al., 2006; Saxena, 2013), only *Cajanus cajanifolius* has been commercialized (Saxena et al., 2005) so far. In order to understand the molecular basis of CMS in pigeonpea mitochondrial genomes have been sequenced (Tuteja et al., 2013). In brief Roche/454 FLX technology together with Sanger sequencing were used to develop one complete (ICPA 2039) and three draft mitochondrial genome assemblies (ICPB 2039, ICPH 2433, and ICPW 29). These sequencing efforts have provided 51 genes, including 34 protein-coding, 14 tRNA and 3 rRNA genes. Comparative analysis among different combinations of four genotypes have provided 18 and 13 chimeric mitochondrial open reading frames (ORFs) in ICPA 2039 when compared with ICPW 29 and ICPB 2039 line respectively. Subsequently, mitochondrial genome was compared with the mitochondrial genomes of 11 other plant species. This has revealed that mitochondrial genome rearrangements has resulted into novel ORFs leading to altered proteins associated to CMS. Tuteja et al. (2013) has also indicated that the mitochondrial genome of pigeonpea shared less number of gene clusters when compared with more distantly related species. Significantly, this study identified 13 potential CMS candidates in the ICPA 2039 from which, five were found to carry parts of other mitochondrial genes and eight were observed to be in the proximity to mitochondrial genes. These outcomes in pigeonpea mitochondrial genome analysis were in accordance with rice. In rice an abnormal mitochondrial *orf79*, CMS line-BoroII encoded a cytotoxic peptide specifically in the microspore which led to male sterility (Wang et al., 2006). In the case of sunflower (*orf552*, Nakai et al., 1995) and sorghum (*orf107*, Tang et al., 1996), such lethal mitochondrial CMS-associated genes have also been reported. Similarly in a recent study, 34 mitochondrial genes were analyzed for expression profiling and sequence variation analysis between CMS line (ICPA 2039) and its maintainer line (ICPB 2039) in pigeonpea. This study showed a possible association of *nad4L* and *nad7* genes with CMS in pigeonpea (Sinha et al., 2015). Further efforts are underway to functionally validate these genes through transformation approaches.

### Fertility Restoration

As described in the previous section, CMS is associated with unusual ORFs in the mitochondrial genome which is generally restored by a fertility restorer (*Rf*) gene encoded by the nucleus (Schnable and Wise, 1998). In many crops including maize and rice, *Rf* genes have been mapped and cloned. As a result, fertility restoration of the hybrids was known to be controlled by one or two genes at two major loci (Cui et al., 1996; Yao et al., 1997). There are many reports that these *Rf* genes are mitochondria-targeting PPR genes encoding pentatricopeptide repeat (PPR)—containing proteins (Small and Peeters, 2000; Akagi et al., 2004). In pigeonpea, A_4_ cytoplasm has been reported to be controlled by two fertility restorer genes (Saxena et al., 2011). Also, the stability of fertility restoration requires the presence of both the dominant genes and the hybrids carrying a single dominant gene were inconsistent with respect to their fertility restoration (Saxena et al., 2011). Few markers associated with fertility restoration have been reported in pigeonpea through linkage mapping and quantitative trait loci (QTL) analysis (Bohra et al., 2012). However, the numbers of markers on linkage maps were less (100–200 SSR markers) and therefore resulted in large genomic intervals under specific QTLs. In this study, three mapping populations (ICPA × 2039 × ICPR 2447, ICPA 2043 × ICPR 3467, and ICPA 2043 × ICPR 2671) were used for fertility restoration phenotyping and SSR genotyping. Single marker analysis (SMA) and composite interval mapping (CIM) were used to detect associated makers with fertility restoration. Using both approaches, 10 markers were found associated with QTLs for fertility restoration. This study identified common markers and consistent QTLs for hybrid breeding in pigeonpea as evident in a number of crop species such as rice (Liu et al., 2004; Ahmadikhah and Karlov, 2006; Wang et al., 2006), maize (Zhang et al., 2006), cotton (Liu et al., 2003), pepper (Ma et al., 2013) and petunia (Bentolia and Hanson, 2001).

### Hybrid Seed Purity Testing

Supply of adequate quantities of pure hybrid seeds to the farmers and maintenance of parental lines of hybrids are other important challenges. Any trace of impurity in the hybrid seeds can substantially affect the productivity, in addition to the purity of the parental lines which is of utmost importance for the success of the hybrid breeding program. Traditionally, breeders perform grow out test (GoT) on representative sample of the seed lot to assess the purity. GoT depends upon several morphological and floral characteristics for determining the purity of seeds which is time consuming and particularly in pigeonpea it requires a season for assessing the purity. In addition, it is also very much labor intensive in comparison to molecular marker based assessment, which saves time and cost. Molecular markers based purity testing of hybrid seeds have provided better options in a number of crops species and are in routine use in many species like rice (Sundaram et al., 2008), maize (Asif et al., 2009), cotton (Ali et al., 2008), safflower (Naresh et al., 2009). Similarly, in pigeonpea SSR based markers have been developed for purity assessment of hybrids and recently a gene based marker for differentiating CMS lines and maintainer lines derived from A4 CMS system developed. In the very first such study, two diagnostic SSR markers were identified for purity assessment in ICPH 2438 (Saxena et al., 2010a). Subsequently, 42 SSR markers for each of the two hybrids (ICPH 2671 and ICPH 2438) have been identified for purity assessment of hybrid seeds (Bohra et al., 2012). Moreover, common markers (CcM0257, CcM1559, CcM1825, and CcM1895) for both hybrids (ICPH 2671 and ICPH 2438) were detected for undertaking multiplex assays. The marker *nad7a_del* derived from *nad7* (gene from mitochondria) differentiated the male sterile line (ICPA 2039) from the fertile line (ICPB 2039). Interestingly, this marker was able to detect as low as 2% admixtures level of ICPB 2039 specific fragment in DNA of ICPA 2039 (Sinha et al., 2015). Recently seven SSR markers (CCB9, HASSR3, HASSR9, HASSR23, HASSR35, HASSR37, and HASSR43) have also been identified for distinguishing the A- line, B- line and hybrid (Bohra et al., 2015).

### Two-Line Hybrid Breeding Systems

Since three line based hybrid technology is technically demanding and incurs cost in commercial hybrid production. These issues have raised significant concerns that led to explore a much simpler technology that would enable accessibility to the farmers growing hybrids in their fields, i.e., two-line hybrid breeding system. Very recently, in pigeonpea a temperature sensitive male sterile line was identified based on field evaluations. This line was developed by crossing a wild relative, *Cajanus sericeus* with a cultivar namely, ICPA 85010 (Saxena, 2014). The perennial nature of the plant and the natural out crossing ability of the crop allows the possibility to evaluate these lines under controlled conditions for their male sterility to fertility transitions with different temperature regimes. These lines could be invaluable for establishing a two-line hybrid system in pigeonpea. Toward this, fertility transition behavior is being studied for more than 20 different combinations of day temperatures, night temperatures, photoperiods, humidity, and light intensities under controlled environment chambers. Preliminary analysis has shown that these lines are responding to day temperature, converting to male sterile with more than 24°C and to male fertile with less than 23°C. In addition, various cytological studies and transcriptome profiling of the male sterile and fertile anthers are also being carried out to identify the putative candidate genes and to understand the molecular mechanism. The identification of candidate gene(s) and/or the trait locus controlling this reversion will play an important role in breeding, ultimately lead to developing a stable two-line system and also making use of elite lines into the hybrid breeding program. Understanding the mechanism will also allow the prediction of performance of F_1_s of the two-line hybrids during different climatic conditions. Transcript profiling and proteomics analysis could be utilized to postulate the possible molecular mechanisms underlying the fertility transition in thermosensitive genic male sterile (TGMS) lines as in case of *Oryza sativa* (Song et al., 2015). A similar approach could also be devised for pigeonpea which will lead to breeding, development and utilization of TGMS lines for a potential two-line hybrid breeding. Breeding of this trait involves identification, cloning and transferring of the major sterility gene. Genetic analysis and fine mapping of this gene has already been carried out in rice (Lee et al., 2005) and wheat (Guo et al., 2006). Using a similar approach, segregating progenies are being developed at ICRISAT to dissect this trait in pigeonpea.

## Outlook

The successes achieved by pigeonpea hybrid breeding will be measured in terms of improvements in livelihood of farmers. However to achieve this daunting task still long way to go for pigeonpea hybrid breeding. The current genomics era has enabled new approaches to long-standing questions as discussed in above sections. A major goal is to assess the current state of research capabilities as the pigeonpea draft genome becomes available and plan systematically future research and extension strategies. It is clear that sequenced genomes have addressed many questions related to developmental biology, genetics and evolution in number of crop species and revolutionized the crops research. Therefore, it is the perfect time in pigeonpea hybrid breeding to learn from past experiences and develop the tools to optimize and fully leverage the value of a sequenced pigeonpea genome. In this regard WGRS based genotyping and array-based SNP genotyping assays (SNP-array) would be helpful in high-resolution mapping of QTLs governing economically important traits such as fertility restoration, biotic and abiotic diseases resistance/tolerance and yield contributing traits etc. In parallel understanding the molecular basis of heterosis, defining heterotic groups, diversification of CMS sources, etc., seems to be potential areas where genomics can be deployed. Further to expand the pigeonpea hybrid area under cultivation, it is highly essential to expand research and development base involving various national programs and public and private seed companies, develop high yielding hybrids for specific agro-ecological regions, fine-tune the hybrid seed production technology for increased efficiency and develop seed certification standards for hybrids and their parents and capacity building in hybrid pigeonpea technology.

### Conflict of Interest Statement

The authors declare that the research was conducted in the absence of any commercial or financial relationships that could be construed as a potential conflict of interest.
